# Human intronic enhancers control distinct sub-domains of *Gli3 *expression during mouse CNS and limb development

**DOI:** 10.1186/1471-213X-10-44

**Published:** 2010-04-28

**Authors:** Amir A Abbasi, Zissis Paparidis, Sajid Malik, Fiona Bangs, Ansgar Schmidt, Sabine Koch, Javier Lopez-Rios, Karl-Heinz Grzeschik

**Affiliations:** 1 Department of Human Genetics, Philipps-Universität Marburg, 35037 Marburg, Germany; 2 National Center for Bioinformatics, Faculty of Biological Sciences, Quaid-i-Azam University, 45320 Islamabad, Pakistan; 3 Department of Animal Sciences, Quaid-i-Azam University, 45320 Islamabad, Pakistan; 4 Biology and Biochemistry Department, University of Bath, Bath, BA2 7AY, UK; 5 Department of Pathology, Philipps-Universität Marburg, 35033 Marburg, Germany; 6 DBM Center for Biomedicine, University of Basel Medical School, Basel, Switzerland

## Abstract

**Background:**

The zinc-finger transcription factor GLI3 is an important mediator of Sonic hedgehog signaling and crucial for patterning of many aspects of the vertebrate body plan. In vertebrates, the mechanism of SHH signal transduction and its action on target genes by means of activating or repressing forms of GLI3 have been studied most extensively during limb development and the specification of the central nervous system. From these studies it has emerged, that *Gli3 *expression must be subject to a tight spatiotemporal regulation. However, the genetic mechanisms and the cis-acting elements controlling the expression of *Gli3 *remained largely unknown.

**Results:**

Here, we demonstrate in chicken and mouse transgenic embryos that human *GLI3*-intronic conserved non-coding sequence elements (CNEs) autonomously control individual aspects of *Gli3 *expression. Their combined action shows many aspects of a *Gli3*-specific pattern of transcriptional activity. In the mouse limb bud, different CNEs enhance *Gli3*-specific expression in evolutionary ancient stylopod and zeugopod versus modern skeletal structures of the autopod. Limb bud specificity is also found in chicken but had not been detected in zebrafish embryos. Three of these elements govern central nervous system specific gene expression during mouse embryogenesis, each targeting a subset of endogenous *Gli3 *transcription sites. Even though fish, birds, and mammals share an ancient repertoire of gene regulatory elements within *Gli3*, the functions of individual enhancers from this catalog have diverged significantly. During evolution, ancient broad-range regulatory elements within *Gli3 *attained higher specificity, critical for patterning of more specialized structures, by abolishing the potential for redundant expression control.

**Conclusion:**

These results not only demonstrate the high level of complexity in the genetic mechanisms controlling *Gli3 *expression, but also reveal the evolutionary significance of *cis*-acting regulatory networks of early developmental regulators in vertebrates.

## Background

Zinc-finger proteins of the GLI family, GLI1, GLI2, and GLI3 act as transcriptional mediators integrating various upstream patterning signals in a context dependent combinatorial and cooperative fashion to direct a multitude of developmental programs. GLI2 and GLI3 can serve both as transcriptional activators or repressors, whereas GLI1, whose expression is transcriptionally regulated by GLI2 and GLI3, appears to play a secondary role, e.g. in potentiating response to the secreted protein sonic hedgehog (SHH) [1].

Mutations in the human *GLI3 *gene cause a variety of dominant developmental syndromes subsumed as "GLI3 morphopathies" [2,3], including Greig cephalopolysyndactyly syndrome (GCPS) [3-5], Pallister Hall syndrome (PHS) [6], postaxial polydactyly type A (PAPA) [7], and preaxial polydactyly type IV (PPD-IV) [2]. Mutations affecting murine *Gli3*, such as extra toes (*Xt*), anterior digit deformity (*add*), and polydactyly Nagoya (*Pdn*), serve as models for GLI3 morphopathies. Mouse embryos with homozygous *Gli3 *deficiency show pleiotropic and lethal congenital malformations with distinct preaxial limb polydactylies [8]. All GLI3 morphopathies show malformations of the autopod, i.e. polydactyly or syndactyly. In addition, craniofacial abnormalities are associated with Greig cephalopolysyndactyly (GCPS), and in the most severe form, Pallister-Hall syndrome (PHS), other developmental malformations occur, such as hypothalamic hamartoma, visceral anomalies, anus atresy, as well as epiglottis and larynx defects [9].

A multitude of studies in mice and other model organisms have suggested that a GLI code, the interplay of the GLI proteins expressed in a quantitatively and temporally fine tuned pattern in adjacent domains, provides a basic morphogenetic tool that is used over and over during embryonic development [1]. GLI-associated patterning has been studied preferentially in the vertebrate central nervous system (CNS) and in limbs. At different rostrocaudal levels of the CNS, dorsoventral neural pattern elaboration can be achieved through the spatiotemporal integration of signals from antagonizing SHH and BMP ligands [10,11] in an interplay with WNT [12,13], fibroblast growth factor [14-16], and retinoic acid signaling [17]. In the vertebrate limb bud, mesenchymal cells aggregate in a proximal to distal sequence to give rise to cartilage condensations that prefigure all limb skeletal components [18]. Sonic hedgehog (SHH) signals direct via GLI transcription factors digit number and identity [19,20]. However, development of proximal skeletal elements (stylopod/zeugopod) is distinctly regulated early during limb-bud formation. Here, GLI3 function independent of SHH signaling appears to be involved [21].

The wide spectrum of tasks demands a tight temporal and spatial regulation of *GLI3 *gene expression and of the proteolytic truncation of activating full length GLI3 protein to a short repressor form, respectively. Whereas the crucial role of hedgehog signal transduction employing the GLI code and the function of downstream target genes have been elucidated by a multitude of studies in humans and model animals [1,20,22], cis-acting sequences and regulatory factors employed in spatiotemporal control of *Gli3/GLI3 *expression remained largely unknown.

Human-fish conserved non-coding sequence elements (CNEs) are candidate *cis*-acting enhancers of gene transcription [23,24]. Previously, we had searched for non-coding sequence conservation between man and pufferfish within the *GLI3 *gene itself and in flanking intervals of > 1 Mb around this gene. In contrast to the situation described for most other genes, ancient conserved non-coding sequence elements are located exclusively in the introns of *GLI3*. Contiguous Human-*Fugu *conservation at this location of human chromosome 7 essentially ends at the limits of *GLI3*, suggesting, that anciently conserved regulatory elements should be located within intronic intervals of this gene.

Indeed, 11 out of 12 *GLI3-*intronic CNEs which show at least 50% identity over a 60 bp window down to *Fugu *acted in transiently transfected cultured cells in a cell type dependent fashion as activators or repressors of reporter gene expression [25,26]. In endogenous *GLI3 *expressing cells the majority of these elements functioned as activators whilst in a GLI3 negative cellular context they actively repressed the transcription. This differential activity was taken as strong evidence in favor of assigning *GLI3*-specific regulatory potential to these CNEs. Two of the CNEs had a repressing potential, even in a GLI3 positive cellular context. The dual nature of a subset of intra-*GLI3 *enhancers could be based on the interaction with different subsets of trans-acting factors (either activators or repressors of transcription) in a cellular context dependent manner, whilst elements with repressing potential, even in a GLI3 positive context, suggest the existence of context independent regulation. In vitro deletion analysis showed that enhancer activity of the CNEs is determined by a combinatorial effect of ancient highly conserved modules and more recent flanking sequences [25,26]. By expressing reporter genes under the control of these human *GLI3*-CNEs in zebrafish embryos, we demonstrated that the activator or repressor function observed in human cell culture was retained *in vivo *in a teleost fish. Only CNEs which could activate reporter gene activity in GLI3 positive context were able to activate a reporter gene in zebrafish embryos. To a large extent, reporter expression induced by these elements coincided with sites of endogenous zebrafish *gli3 *expression; however, there was considerable redundancy in expression control by the different CNEs [25,26]. In transgenic mice assay, we could show that CNE2, an ultraconserved sequence element within intron 2 of GLI3, enhanced reporter gene expression at sites of endogenous *Gli3 *expression [26]. For CNE2, a similar mouse expression pattern was reported in a genome-wide enhancer test of non-exonic ultraconserved elements in transgenic mouse embryos (element #111, [27]). In the genome-wide enhancer test two further enhancers were identified within *GLI3*, element #1213 encasing CNE1, previously identified by Abbasi et al. [25], and the novel element #1586 http://enhancer.lbl.gov. It is of notice that in that screen a candidate enhancer element flanking *GLI3 *in an upstream position was unable to activate reporter gene expression (element #1132) Reporter elements with putative enhancers encasing CNE1, CNE2, and CNE3 activated reporter gene expression in the dorsal spinal cord at sites of endogenous *Gli3 *expression when tested by chicken *in ovo *electroporation [12].

In this study, we employed transgenic assays to show that *GLI3 *intronic CNEs, which are able to activate transcription in cell cultures and zebrafish, can induce reporter gene activation at sites of endogenous *Gli3 *expression also in chicken and mice. Reporter gene expression was identified in craniofacial structures, limbs, brain, spinal cord, eye, and gut. In particular, these enhancers were able to target transgene expression to many known regions of endogenous *Gli3 *transcription in limb buds as well as along the anterior-posterior and dorsal-ventral axis of the developing mouse neural tube.

## Results and Discussion

### Tetrapod-teleost conserved GLI3-intronic enhancers identified by comparative sequence analysis

**Figure 1:**
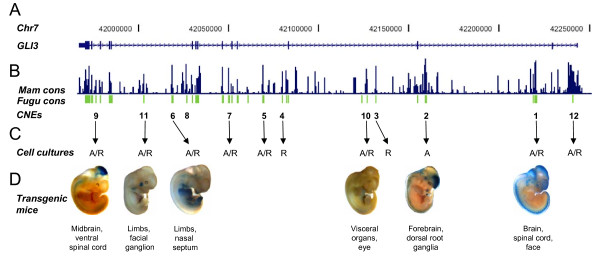
**GLI3 specific, spatiotemporal pattern of reporter gene expression in transgenic mouse embryos is evoked by intronic conserved non-coding sequence elements (CNEs) acting in a complementary fashion**. (A) Human chromosome 7 coordinates along with the graphical representation of exons (numbered) and introns of *GLI3*. (B) Graphical plot depicting evolutionary conservation of human *GLI3 *across multiple mammalian vertebrates (blue) and *Fugu *(green) generated using sequence alignment tools in the UCSC comparative genomics alignment pipeline. http://genome.ucsc.edu. (C) 1-12: Human/*Fugu *CNEs characterized as enhancers through functional assays by employing human cell lines and zebrafish embryos [26,28]. A, R: activating or repressory potential of these enhancers in cell culture. (D) Subset of 6 intronic CNEs whose spatiotemporal regulatory potential is defined in transgenic mouse assay. Selected embryos are shown at representative time points (E11.5 or E12.5) along with their primary target sites.

Multi-species alignment of human *GLI3 *genomic sequence with orthologous intervals from other vertebrate species localized 12 intronic conserved non-coding elements, showing at least 50% identity over a 60 bp window down to *Fugu*. These elements are distributed across almost the entire *GLI3 *interval (Figure 1A and 1B), with 2 elements in each of introns 2, 3, 4, and 10 and one in each of introns 1, 6, and 13 [25]. The *GLI3 *specific gene regulatory functions of 11 of these putative human enhancers had previously been determined using human cell lines (Figure 1C). The elements which could activate reporter gene expression in cell cultures functioned likewise in zebrafish embryos [25]. Additionally, the spatiotemporal aspects of one ultraconserved element, CNE2, were analyzed in mouse embryos [26]. However, the spatiotemporal functionality of other *GLI3 *associated enhancers in a mammalian model remained to be defined.

To determine the tissue specific role of CNEs 1, 2, 6, 9, 10, and 11, which had acted as enhancers in cell cultures and zebrafish embryos, we generated transgenic mice driving *lacZ *reporter gene expression under the control of each CNE (Figure 1 and Additional file 1: Table S1). The boundaries of the selected subset of enhancer regions were defined bearing in mind that full scale enhancer activity is determined by a combination of core sequences conserved between human and teleosts *(Fugu) *and flanking tetrapod-specific sequences [28].

CNE10 mediated *lacZ *expression was largely confined to foregut derivatives, eye and mammary placodes, and will be dealt with in detail elsewhere. Here, we focus on the potential of CNEs 1, 2, 6, 9, and 11 to replicate endogenous *Gli3 *expression pattern during development of the limbs and the central nervous system (CNS).

### Enhancer elements from *GLI3 *introns directing expression in the mouse limb bud

**Figure 2:**
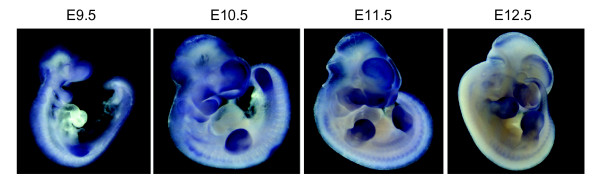
**Detection of Gli3 expression by whole mount *in situ *hybridization at different time points in mouse embryonic development**. Standard procedures were employed.

The Gli3 expression patterns within the nascent limb bud are highly dynamic (Figure 2). Initially, Gli3 is expressed broadly in the mesenchyme of the emerging limb bud. At later stages, the genetic antagonism between Gli3 and Shh results in exclusion of the Gli3 expression domain from the posterior limb mesenchyme [29].

This expression pattern is in agreement with limb specific anomalies in Gli3 mutants, in particular the anteroposterior patterning of distal limb elements, i.e. the autopod [30]. Gli3 likewise plays a critical role in regulating the patterning of proximal and intermediate skeletal elements of limbs (stylopod/zeugopod patterning) at very early stages of development [21]. Importantly, the Gli3 functions in stylopod/zeugopod skeletal patterning are independent of its role in the anteroposterior patterning of the handplate [21]. However, as Gli3 is broadly present throughout the developing limb (Figure 2), its expression within proximal mesenchymal condensations (cartilage condensations of stylopod and zeugopod elements) can easily be overlooked [21]. Nevertheless, recently, through Western analysis and in situ hybridization, Gli3 was detected in the cartilage of developing limb elements [31] and plays a critical role in regulating proliferation during endochondral bone formation [32].

**Figure 3:**
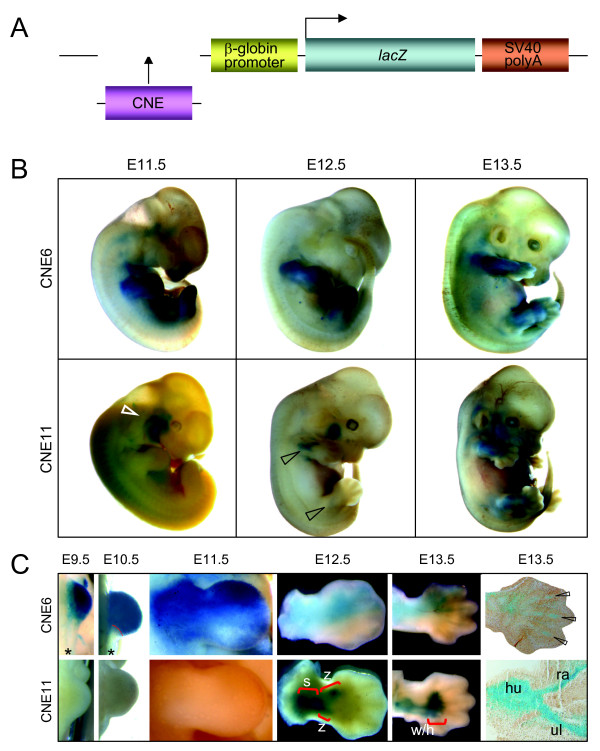
**Enhancers CNE6 and CNE11 govern distinct aspects of *Gli3 *expression in developing limbs**. (A) Diagram of the *lacZ *reporter gene construct employed to test the enhancer activity of GLI3-CNEs in mouse embryos. (B) Whole mount views of transgenic mouse embryos at days E11.5, E12.5, and E13.5 expressing *lacZ *under control of CNE6 or CNE11. White and black open arrowheads indicate reporter expression within facial (VII) nerve and proximal limb elements, respectively. (C) Developing mouse forelimbs from embryonic day E9.5 to E13.5 showing domains of reporter gene activity induced by CNE6 (upper row) and CNE11 (lower row). CNE6-directed *lac*Z expression starts early, coinciding with the emergence of the limb bud, and is largely confined to anterior aspects. CNE11 induced reporter expression starts at E12.5 and is restricted to mesenchymal condensations of proximal elements (stylopod and zeugopod, brackets s and z). In E13.5 limbs, CNE11 governed expression extends to the proximal portion of handplate (bracket: w wrist, h handplate) but not into the digits. Longitudinal sections through the E13.5 forelimbs show CNE6-directed reporter expression within distal skeletal elements (digits, open arrowheads) and CNE11-governed X-gal staining in prospective humerus (hu), radius (ra) and ulna (ul). Asterisk symbol shows posterior margin of E9.5 and E10.5 forelimb buds devoid of reporter expression.

In transgenic mouse embryos, the spatiotemporal regulatory activity of two distinct enhancers, CNE6 and CNE11, reflects several of the known aspects of endogenous *Gli3 *expression within cartilaginous and non cartilaginous mesenchyme of embryonic limbs (Figure 3). CNE6-directed *lacZ *expression coincides with the emergence of the limb bud, continues towards anterior, and concentrates at E13.5 at the prospective mesenchyme condensations in the digits (Figure 3B and 3C). This spatiotemporal activity overlaps with Gli3 function during formation of proximal skeletal elements, stylopod/zeugopod [33].

In contrast, CNE11 directs reporter expression specifically within proximal regions of the limb bud from stage E12 on, once the mesenchyme starts to condense and form precartilage (Figure 3B and 3C). This spatiotemporal activity overlaps with Gli3 function in patterning of proximal skeletal elements, stylopod/zeugopod [21]. Thus CNE6 and CNE11 elements showed non-redundant regulatory activities. Since the sites of stable transgene insertion in the mouse lines have not been determined, this conclusion awaits confirmation by a larger number of independent transgenic mouse embryos. Multiple sequence alignment coupled with pattern recognition computer programs identified conserved binding sites for number of transcription factors in CNE6 and CNE11 intervals (see Additional file 2 & Additional file 3: Figures S1 & S2), many of which are among the core set of limb regulators and are known to be co-expressed with Gli3 during early limb patterning and growth [34]. Their role in the control of *Gli3 *expression by interaction with CNE6 and CNE11 will be tested experimentally.

It is of note that *Gli3 *transcriptional activity appears to be important for the separation of individual fingers [2], and also endogenous *Gli3 *is known to be expressed strongly in the interdigital mesenchyme at E12.5 (Figure 2 and [35]). However, the mesenchyme in between the prospective digit rays is an area where we did not observe this much expression with both CNE11 and CNE6 enhancer elements. Previously, we had detected reporter gene activity in the interdigital mesenchyme at E13.5 in transgenic mice employing the CNE2 enhancer element [26]. Earlier interdigital Gli3 expression appears to be governed by other enhancer regions. The VISTA enhancer browser http://enhancer.lbl.gov lists as element_1585 a Gli3 intragenic enhancer activating reporter gene activity at E11.5 distally in mouse embryo limbs. Study of the temporal and spatial activity of this enhancer element, which did not meet the inclusion criteria of our study, must be awaited to determine if it acts in a complementary fashion or if it overlaps the activity of CNE6 or CNE11.

### Enhancer elements from *GLI3 *introns directing expression in the chicken limb bud

**Figure 4:**
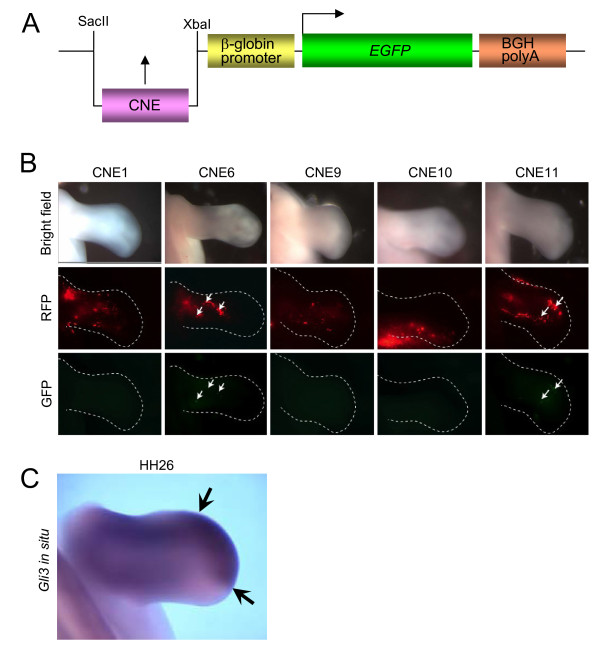
**Limb tissue specificity of GLI3-CNE enhancers is maintained in chicken limb buds**. (A) Diagram of the reporter construct employed to test the enhancer activity of CNEs in chicken embryos. The β-globin promoter was used to drive the GFP expression. The CNEs were cloned in SacII-XbaI sites. (B) Whole mount of limb buds 48 hours after co-electroporation of enhancer construct and RFP at stage HH20 analyzed simultaneously for expression of a control RFP expression vector and a GFP reporter under control of one out of five CNEs. Top row: Control bright field view of electroporated limbs; middle row: RFP fluorescence; bottom row: GFP fluorescence. Electroporated limbs showing GFP signals in addition to RFP fluorescence: CNE1 n = 0/5, CNE6 n = 3/12, CNE9 n = 0/7, CNE10 n = 0/2, CNE11 n = 4/6. Consistent with reporter expression data from mice, only CNE11 and CNE6 enhancers drove GFP reporter expression in developing chicken limbs. Arrows: GFP expressing regions at embryonic stage HH26. Anterior to the top. (C) Distribution of *Gli3 *transcripts shown via *in situ *hybridisation. At stage HH26 transcripts are seen at the distal tip (arrows) and more proximally, but absent from the distal posterior region. Note that GFP expressing cells in 4B are found in a region of the limb where *Gli3 *appears to be expressed. Anterior to the top.

Expressing a reporter controlled by human *GLI3*-CNEs in transgenic zebrafish embryos could not identify one of them reliably as fin-specific enhancer [28]. To determine, if limb bud specificity similar to the results obtained in mice was attributed to individual *GLI3*-CNEs in birds, we analysed if CNEs 1, 6, 9, 10, and 11 act as enhancers of *Gli3*-specific expression in the chicken limb buds. These CNEs were cloned into a GFP reporter construct under the control of a β-globin promoter (Figure 4A)[36] and co-electroporated together with an RFP reporter, to control for electroporation efficiency, into the chicken wing bud at stage HH 19/20. The developing chicken limbs were assessed for RFP and GFP expression 48 hours following electroporation when the embryos had reached approximately stage HH 26. At this stage, Gli3 has been reported to be expressed throughout the proximal region and at the distal anterior edge of the chicken wing bud (Figure 4C) [37]. Figure 4B shows the results of the electroporation experiments. The upper row shows control bright field photographs of the electroporated limb buds analyzed in the next two rows. The middle row shows RFP expression in the limb bud indicating the extent of electroporation. The bottom row shows GFP expression in the same limb bud. CNEs 1, 9, and 10 gave no GFP expression despite RFP being expressed throughout the limb. CNE6 and CNE11 had weak GFP expression in 3/12 and 2/6 cases, respectively, indicating slight enhancer activity. The distribution of *Gli3 *mRNA in the limb at stage HH26 is shown below via *in situ *hybridisation (Figure 4C). Gli3 highly expressed distally but also proximally at the posterior margin and therefore reporter activity appears to be within the region of the limb expressing Gli3. At earlier stages, Gli3 is more highly expressed throughout the anterior of the limb bud, and therefore we might expect that electroporation of the putative enhancer constructs at an earlier stage would provide a better test for enhancer activity. In a second set of experiments we therefore electroporated CNE11 into the presumptive limb mesenchyme [38] at stage HH14 of 5 embryos, and then looked for enhancer activity 48 hours post electroporation at approximately stage HH23. RFP expression was found throughout the anterior region of the limb, however, no GFP expression was seen in any of the cases examined (data not shown), although the construct had been successfully electroporated into the region of the limb bud, which would be expressing *Gli3*.

Consistent with data from mice, CNE11 and CNE6 were able to drive reporter expression in developing chicken limbs at stage HH26 (Figure 4B, arrows), while CNEs 1, 9, and 10 were not. We have previously demonstrated that conserved non-coding elements downstream of the homeobox gene SHOX have enhancer activity using the same assay [36]. In that case three out of the eight CNEs tested showed enhancer activity indicating that some but not all conserved non-coding elements act as enhancers. The remaining CNEs may act to regulate the gene in another way, for example, as repressors. Another difference between the present study and previous experiments with SHOX is that Gli3 has a more restricted expression pattern and lower level of expression than SHOX at later stages. This may reduce the chance of introducing a putative enhancer construct into a Gli3 expressing region of the limb bud and therefore make it more difficult to detect enhancer activity. However, introduction of CNE11 into the limb at an earlier stage when Gli3 expression is more widespread did not show any enhancer activity. This observation is in line with the data obtained in mouse embryos where CNE11 started to enhance reporter gene activity at E12.5 and was inactive at earlier stages.

### *GLI3 *enhancer activity reflects evolutionary advances of limb specification

Our finding that enhancer elements within *GLI3 *act differentially during mouse limb patterning corroborates the current view of limb evolution. Despite the morphological and functional diversity of fish fin and mammalian limbs, development of these structures is regulated by a similar and related set of genes [39]. Evolution of regulatory components was proposed to be key for the origin and subsequent morphological diversification of the vertebrate fin/limb skeleton [40]. The spatiotemporal regulatory role of CNEs 6 and 11 in zebrafish was more redundant with that of other GLI3 enhancers and not preferentially used for fin/limb patterning as now seen in mice [26,28]. During early embryonic development of the tetrapod limb, GLI3 plays a double role: SHH dependent anteroposterior patterning of the autopod and SHH independent specification of skeletal elements along the proximodistal axis from the stylopod up to the distal margin of the zeugopod [19,21]. In accordance with these two distinct roles, this study has defined distinct enhancer regions residing in introns of *GLI*3 that independently regulate expression in the evolutionary ancient stylopod and zeugopod or in modern skeletal structures of the limb autopod, respectively. This suggests that redeployment of ancient *cis*-regulatory elements to direct *GLI3 *expression in distinct limb domains might have been instrumental in diversifying the vertebrate limb skeleton during the course of evolution.

### Enhancer elements from *GLI3 *introns directing expression in the mouse CNS

**Figure 5:**
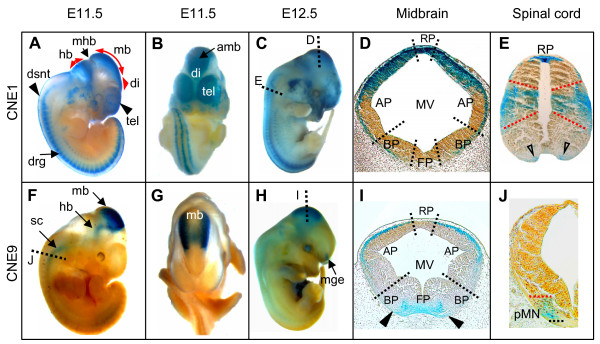
**CNE1 and CNE9 govern *lacZ *expression along distinct domains of brain and spinal cord**. (A-C) Whole mount views of transgenic mouse embryos expressing the reporter under control of CNE1 at E11.5 (A, B), and E12.5 (C). (F-H) Whole mount views of embryos carrying CNE9 as enhancer of *lacZ *expression at E11.5 (F-G), and E12.5 (H). (D, I) Transverse sections through the midbrain at the level shown with dotted lines in panels (C) and (H). (D) In the roofplate and dorsolateral part of alar-column of midbrain the CNE1-induced expression is apparent in marginal, mantle, and ependymal layers of neuroepithelium, whereas in medial section of alar- plate/entire basal-plate of midbrain, expression is restricted to the marginal layer. (I) CNE9-driven *lacZ *expression is present in ventral midline of caudal midbrain, whereas dorsally reporter signal is confined to the dorso-lateral marginal tissue. (E, J) Transverse sections through the spinal cord at the levels shown with dotted lines in the panels (C) and (F). (E) CNE1-generated transgene expression in the spinal cord is confined to the roofplate (RP), progenitors of Dp5, Dp6, Vp0, Vp1 interneurons, and progenitors of V3 interneurons (open arrowheads). (J) CNE9-induced lacZ expression in the spinal cord was present up-to embryonic day E11.5 and was confined to progenitors of motor neurons (pMN). amb, anterior midbrain; drg, dorsal root ganglia; dsnt, dorsal neural tube; hb, hindbrain; di, diencephalon; mb, midbrain; mge, medial ganglionic eminence; mhb, midbrain-hindbrain boundary; sc, spinal cord; tel, telencephalon. MV, mesencephalic vesicle; AP, alar-plate; BP, basal-plate; FP, floorplate.

The spatiotemporal activities of CNE1, CNE2, and CNE9 complemented each other in the control of reporter expression reflecting part of the GLI3-specific pattern in the brain, spinal cord and craniofacial structures (Figure 1D, Figure 5). The mouse embryo expression patterns governed at E11.5 by CNE1 and CNE2, respectively, are independently reported in the Vista enhancer browser for sequence elements 1213 and 111 which include the sequences employed here http://enhancer.lbl.gov, adding credibility to the notion that the CNEs studied are bona fide *GLI3 *enhancers.

At day E11.5, CNE1-controlled *lacZ *was strongly expressed in the dorsal brain and spinal cord, and less prominently in hypaxial buds of the thoracic somites, proximal muscle masses in the forelimb bud, dorsal root ganglion, and in the facial mesenchyme (Figure 5A &5B). At E12.5, stronger reporter activity was seen in the cerebellum and nerves innervating the dorsolateral trunk region and forelimbs, and extended more rostrally in the head mesenchyme (Figure 5C). In the midbrain, transgene expression was present in the roofplate, dorso-lateral portion of the alar plate, and confined to a marginal layer of the basal plate (Figure 5D). In the spinal cord, X-gal signal was present in the roofplate, in a central region presumably covering the progenitor domains of dorsal interneurons dl5-dl6 and the ventro-lateral progenitor domains Vp0-Vp1, as well as in the ventral most mantle zone of the spinal cord occupying the post-mitotic V3 interneurons (Figure 5E). Additionally, *lacZ *activity was detected in the medial and lateral nasal processes, precartilage primordium of nasal capsule, Meckel's cartilage, lateral palatine process, and in the dental lamina (data not shown). The role of CNE2, a highly conserved non-coding element, has been outlined previously [26]. Whereas the reporter expression driven by CNE2 was present throughout the walls of telencephalic vesicle, CNE1 activity was confined medially. Thus, there is a partial overlap in the activities of these two enhancers within the anterior domain of the forebrain. At E11.5, reporter activity induced by CNE9 was detected in the dorso-lateral aspects of the anterior and posterior midbrain regions and in ventral portions of the hindbrain and spinal cord up-to the level of forelimb region (Figure 5F and 5G, arrows). At E12.5, CNE9 driven transgene expression was also demonstrated in the medial ganglionic eminence (Figure 5H, arrow). In transverse sections, CNE9 driven *lacZ *expression was observed in the ventral midline of caudal midbrain (presumptive dopaminergic neurons, Figure 5I, arrow heads) and confined to the dorso-lateral marginal layer (Figure 5I). Reporter activity induced by CNE9 in the CNS overlapped with CNE1 only in the dorso-lateral marginal tissue of the midbrain (Figure 5I).

Notably, in the developing spinal cord CNE9 induced reporter expression appeared up to E11.5 in the motor neuron progenitor domain (pMN, Figure 5J). Thus, regulatory factors operating at different time intervals via CNE9 or CNE1 could activate *GLI3 *expression in motor neuron or interneuron territories, respectively.

According to current models, in the ventral spinal cord positional information encoded by a ventral to dorsal SHH gradient is transmitted by a GLI code, the interplay of activator or repressor functions of GLI proteins (reviewed by [41]). In mouse embryos, GLI1 functioning as transcriptional activator is expressed in the ventral neural tube whereas the expression pattern of *Gli2 *remains uniform along the dorsal-ventral axis of neural tube [42,43]. *Gli3 *is expressed extensively in the intermediate and dorsal spinal cord regions. Consistent with its expression pattern, genetic studies with mice suggest that Gli3 repressor activity (proteolytically processed isoform) is essential for the normal patterning of at least six neuronal classes: V2, V1, V0, dI6, dI5, and dI4 neurons in the intermediate region of the spinal cord [29]. In addition to its repressor role in the intermediate spinal cord, Gli3 can transduce Hedgehog signaling as an activator. For instance, at the highest levels of Hh (ventral most region of spinal cord) all expression of the Hh target gene Gli1 is dependent on both Gli2 and Gli3. Unlike Gli2, however, Gli3 requires endogenous Gli1 for induction of floor plate and V3 interneurons [44]. Beyond the well established dorso-ventral patterning function through a Gli3-derepression mechanism, Shh and Gli3 activities are required to promote the timely appearance of motor neuron progenitors (MN) in the developing spinal cord [44,45]. The weak activator functions of endogenous Gli3 observed by Bai and coworkers [44] near the source of Shh are compatible with a subtle expression of Gli3 protein in the three most ventral domains, FP, V3 and MN. The domains of *Gli3 *expression and Gli3 function in the developing spinal cord reported in these studies are mirrored perfectly by the sites of CNE1 and CNE9 action.

Electroporation of reporter constructs employing conserved *Gli3*-intronic sequences, which include CNE1 or CNE2, induced the strongest expression signals preferentially in the dorsal spinal cord [12]. However, time, amount, and location of expression governed by these enhancer elements, are analyzed in greater detail in transgenic mouse embryos.

Evolutionary conserved transcription factor binding sites (TFBSs) are predicted in CNE1, CNE2, and CNE9 intervals for multiple established developmental regulators (see Additional file 4 & Additional file 5: Figures S3 & S4, and [26]), many of which are known to be co-expressed with *Gli3 *during embryonic development of brain and spinal cord [34]. Their interaction with these enhancers remains to be determined experimentally.

Including CNE2, we have identified three independent *GLI3*-intronic enhancer regions that control reporter expression in developing neural tissues of the mouse embryo in a time- and position-specific complementary fashion. With multiple independent enhancers controlling early CNS patterning, Gli3 follows suit other key developmental genes with a high level of complexity in their genetic regulatory mechanisms governing neural tube patterning [12,46,47].

### Multiple independently acting regulatory sequences herald the occurrence of higher levels of modularity in the body plans of modern vertebrates

**Figure 6:**
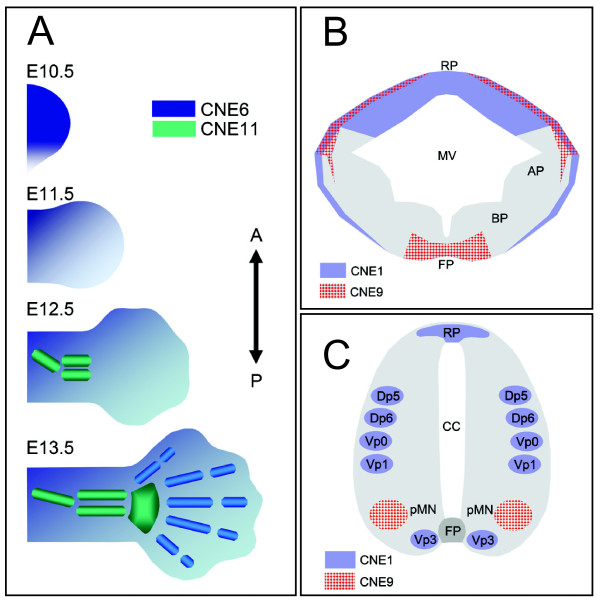
**Limb or CNS specific enhancers show complementary regulatory potential reflecting endogenous GLI3 expression**. (A) Diagrams summarizing reporter signals of two independent enhancers, CNE6 and CNE11, which regulate expression distinctly within evolutionary ancient (stylopod, zeugopod) and modern aspects (autopod) of mammalian limb. At E10.5 and E11.5, CNE6-governed reporter activity (blue color) was detected throughout the developing limb bud, with the exception of the posterior margin. At these time points no CNE11 activity was seen in the limb bud. By E12.5, CNE11 induced transgene expression specifically within precartilage condensations of mesenchyme within presumptive proximal limb elements (green). At this time point, CNE6 activity was confined to digital rays, digital inter-zones, and to the non-cartilaginous mesenchyme encasing the precartilage condensations of proximal and distal limb elements. By E13.5, when the precartilage condensations of mesenchyme are replaced by cartilage, CNE11 activity was retained in the stylopod and zeugopod, and was extended more distally up-to cartilaginous elements of digit arch (wrist/ankle). At this time point, CNE6 directed reporter expression was focused on the individual digits. A, anterior; P, posterior. (B, C) Diagrams summarizing enhancer activities of CNE1 (lavender) and CNE9 (red) along dorsal-ventral aspects of midbrain (*B*) and spinal cord (*C*). CNE1- and CNE9-directed expression during CNS development is non-overlapping except in the dorso-lateral marginal neuronal tissue of the midbrain. RP, roofplate; FP, floorplate; pMN, progenitor area of motor neurons; Dp/Vp, progenitors of dorsal/ventral interneurons; AP, alar plate; BP, basal plate; MV, mesencephalic vesicle; CC, central canal.

It has widely been accepted that differences in morphological and anatomical traits among closely related species are correlated to changes in *cis*-acting sequences [48]. Our study on the spatiotemporal activity of independent, anciently conserved *cis*-regulatory modules, controlling expression of the evolutionary conserved developmental regulator gene *GLI3 *during limb (Figure 6A) and CNS patterning (Figure 6B and 6C), suggests that these enhancers dictate expression in discrete developmental compartments. Above that, cellular subpopulations within a given compartment, such as the motor neuron or interneuron territories in the spinal cord behave as semiautonomous units with respect to expression control of *GLI3 *(Figure 6). This subtle specification of enhancer functions corroborates the view that *cis*-acting regulatory networks of early developmental regulators are often modular, with multiple independent enhancers mediating the expression of associated gene in multiple embryonic compartments independently [49,50]. Functional changes in one specific *cis*-regulator through mutations might alter the spatiotemporal distribution of the associated gene product in one developmental domain, whereas the rest of the expression pattern and the protein activity will largely remain un-interrupted. Thus changes in *cis-*acting sequences will have minimal cost on overall fitness and can serve as raw material for the evolution of morphological and anatomical diversification within and between species.

## Conclusion

This work adds to the growing body of data indicating that cell fate and tissue patterning during embryonic development are governed through the temporal integration of different combinations of signaling ligands at autonomous enhancers. A growing body of empirical evidence suggests that it is not uncommon for developmental regulatory genes to harbor their entire or subset of *cis*-acting gene regulatory elements within their intronic intervals http://condor.fugu.biology.qmul.ac.uk and http://enhancer.lbl.gov. The location of ancient *Gli3*-specific enhancers exclusively within the gene hints at a their critical relevance, since genomic rearrangements during evolution leaving the gene intact would not have separated *cis*-acting regulatory elements from the coding sequence.

Our description of a catalog for *GLI3 *specific cis-regulatory sequences offers a new perspective for studying the genetic mechanisms by which the downstream effectors of hedgehog signaling cascade might themselves be regulated at correct place and precise time to direct pattern formation along the body axis during embryogenesis. Identifying the code of *trans*-acting molecules which jointly activate specific *GLI3 *enhancers, such as CNE11 in stylopod and zeugopod or CNE6 in the autopod may help to understand the mechanisms by which a proper balance between SHH and GLI3 transcripts is established in complementary domains of the limb bud.

In humans, functional deficiency of GLI3 is associated with polydactyly or craniofacial abnormalities [9]. Mutations in enhancers directing *GLI3 *expression in the affected developmental fields, such as CNE1 or CNE6, can potentially affect the timely availability of *GLI3 *transcript during embryogenesis. They are novel targets for mutation analysis in patients with GLI3 morphopathies which cannot be attributed to a mutation in the coding sequence of this gene.

## Methods

### Reporter constructs

Highly conserved sequence elements from GLI3 introns (CNE1, 6, 9, 10, 11) (Figure 1) were chosen as candidate enhancer sequences and PCR amplified using a high-fidelity DNA polymerase (Herculase^®^, Stratagene) with primers containing restriction site tags [26,28], inserted into the p1230 vector (a generous gift of R. Krumlauf) in front of the human β-globin minimal promoter driving a *lacZ *reporter gene [51], and cloned in Top10 bacteria (Invitrogen) using standard technology. Purified plasmids were controlled for correctness of insert sequences by automated sequencing (ABI 377, Applied Biosystems). The nucleotide sequences, extent of human-fish conservation, genomic coordinates, of selected subset of human CNEs (CNE1, 6, 9, 10, 11) are provided (see Additional file 1 & Additional file 6: Table S1 & Dataset S1).

### Establishing transgenic mice

Inserts were separated from vector sequences as described previously [26,28] and diluted for injection into CB6F2 or FVB/N zygotes in 10 mM Tris, pH 7.5, 0.1 mM EDTA, pH 8.0 buffer in a final concentration of 1-3 μg ml^-1^. Injected oozytes were transferred by PolyGene AG Rümlang, Switzerland, or IMT Transgenic Mouse Unit, Philipps Universität Marburg, Germany, into the oviduct of foster mice. The amount of DNA applied cannot be determined with certainty, but it is estimated that 1-2 pl are microinjected into each male pronucleus of fertilized eggs. G_0 _embryos were allowed to develop to term, and by using genomic DNA (extracted from tail or ear tissue using standard protocols) at least 3 offspring carrying recombinant constructs were identified by PCR (primers: "XgalF", 5'-CAACAGTTGCGCAGCCTGAATG-3'; "XgalR", 5'-GTGGGAACAAACGGCGGATTG-3') and used for breeding with the respective wild type animals. Transgenic males were subsequently used as studs with wild type females to maintain transgenic lines and to generate embryos of different age for lacZ expression analysis. Experiments with mice were approved by the appropriate governmental authority "Regierungspräsidium Giessen", Reg. No.: V54-19c20/15cMr20/5. Mice were housed and maintained in the Central Animal Facility of the Medical Faculty of the Philipps-University Marburg according to approved institutional guidelines. The number of transgenic mouse lines established for each CNE, and the number of independent lines showing consistent expression pattern are provided in Additional file 1: Table S1.

### Whole mount mouse embryo preparation and histological analysis

Stable expression of the *lacZ*-reporter gene in transgenic mouse embryos under the control of a beta-globin minimal promoter enhanced by each of the respective CNEs was inspected at different time points in development after whole mount X-gal staining and on histological sections. Time of gestation was calculated taking noon of the day of detection of a vaginal plug as embryonic day 0.5 (E 0.5). Embryos were harvested at approximately E9.5, 10.5, 11.5, 12.5, and 13.5, dissected free of extraembryonic membranes (which were retained for control of transgene insertion), fixed in 0.5% glutaraldehyde at 4°C for 30' to 2 hours, depending on their developmental stage, washed with PBS (containing 2 mM MgCl_2_), and stained overnight in X-gal reaction buffer [35 mM K_3_Fe(CN)_6_, 35 mM K_4_Fe(CN)_6_, 2 mM MgCl_2_] containing 0.1% X-gal at 37°C. The staining reaction was stopped by washing the embryos repeatedly in PBS. The embryos were postfixed overnight in 0.5% glutaraldehyde at 4°C. To analyze the distribution of reporter gene-expressing cells, embryos were dehydrated, embedded in paraffin wax, sectioned at 10-40 μm, deparaffinized, mounted for histological analysis, following standard protocols.

### Chicken in ovo electroporations and enhancer reporter expression analysis in chicken limb buds

Fertilized White Leghorn eggs were obtained from H. Stewart (Lincolnshire, U.K.) and incubated at 39°C. 1 μg/μl of GFP reporter construct containing a selected CNE was co-electroporated with 1 μg/μl of RFP expression vector (RFP in pCAGGs driven by the U6 promoter from chick chromosome 28 and 0.02% fast green [52]. This mix was injected into the limb bud mesenchyme at stages HH 19/20 and electroporated with 1 pulse of a square wave current generated by a CUY21 Bex Company electroporator (Tokyo, Japan) at 45 volts for 50 msec using 3 mm platinum electrodes placed anterior and posterior to the limb bud. Limb buds were analyzed as whole mounts for GFP and RFP expression 48 hours following electroporation when the embryos had reached approximately stage HH 26 using a UV fluorescence dissecting microscope and a GFP or TXR filter respectively.

### Bioinformatics based analysis

Approximately 276 kb of human *GLI3 *genomic interval (7p14.1) was compared with orthologous sequences from multiple placental mammalian species (22 species) by using the PhyloP [53] and also with the *Fugu gli3 *by using chain and net alignments [54] available at UCSC genome browser http://genome.ucsc.edu. Human *GLI3 *genomic sequence was used as baseline and annotated by using exon/intron information available at UCSC.

Within human CNE1, CNE6, CNE9 and CNE11 genomic intervals the conserved transcription factor binding sites were identified with rVISTA. 2.0 http://rvista.dcode.org[55] searches against the collection of 500 vertebrate TF matrices from the TRANSFAC library, with matrix similarity cuttoff 0.85. For better representation, the conserved putative TFBSs were manually overlaid on CustalW2 [56] derived multiple sequence alignments.

## Authors' contributions

Wet lab experiments were designed and performed by K-HG, AAA, ZP, JL-R, SM, and FB with advice in histological analysis by AS, and SK. Computational analyses were performed by AAA. The manuscript was written by K-HG and AAA. All authors read and approved the final publication.

## Supplementary Material

Additional file 1 Table S1: Tetrapod-Teleost Conserved Non-Coding elements (CNEs) from Introns of Human *GLI3 *Selected for Functional Analysis in Transgenic mice assay. Click here for file

Additional file 2 **Figure S1: ClustalW-derived multiple alignment of CNE6 sequence across a diverse set of mammalian species**. Star symbols underneath represent nucleotide positions conserved in all species. Conserved putative transcription factor binding sites (TFBSs) are enclosed in rectangles. ALX4, aristaless-like homeobox 4; SOX5, SRY (sex determining region Y)-box 5; PITX2, paired-like homeodomain transcription factor 2; LHX3, LIM homeobox protein 3; HOXD13, homeobox D13; PITX1, paired-like homeodomain transcription factor 1; GLI, GLI family zinc finger; HOXD11, homeobox D11; HOXA7, homeobox A7; dHAND, basic helix-loop-helix transcription factor; MSX1, msh homeobox 1; PBX1, pre-B-cell leukemia homeobox 1. Click here for file

Additional file 3 **Figure S2: ClustalW-derived multiple alignment of CNE11 sequence across a diverse set of mammalian species.** Star symbols underneath represent nucleotide positions conserved in all species. Conserved putative transcription factor binding sites (TFBSs) are enclosed in rectangles. HOXA13, homeobox 13; HOXD13, homeobox 13; dHAND, basic helix-loop-helix transcription factor; TBX3, T-box 3; HOXA3, homeobox 3; PITX2, paired-like homeodomain transcription factor 2. Click here for file

Additional file 4 **Figure S3: ClustalW-derived multiple alignment of CNE1 sequence across a diverse set of amniotic vertebrate species.** Star symbols underneath represent nucleotide positions conserved in all species. Conserved putative transcription factor binding sites (TFBSs) are enclosed in rectangles. MEIS1, Meis homeobox 1; PAX3, paired box 3; FOXM1, forkhead box M1; SOX5, SRY (sex determining region Y)-box 5; HOXA4, homeobox 4; FOXP3, forkhead box P3; dHAND, basic helix-loop-helix transcription factor; MSX1, msh homeobox 1; HOXA3, homeobox 3; NFKB1, nuclear factor kappa-B; GATA3, GATA binding protein 3; CDX2, caudal type homeobox 2; OCT4, POU domain, class 5, transcription factor1; TBX5, T-box 5; CHX10, visual system homeobox 2; SP1, Sp1 transcription factor; PBX1, pre-B-cell leukemia homeobox 1; TCF. Transcription factor; LEF, lymphoid enhancer binding factor. Click here for file

Additional file 5 **Figure S4: ClustalW-derived multiple alignment across a diverse set of amniotic vertebrate species reveals highly conserved putative TFBSs within the core of the CNE9 interval.** Star symbols underneath represent nucleotide positions conserved in all species. Conserved putative transcription factor binding sites are enclosed in rectangles. HOXA3, homeobox 3; TBX5, T-box 5; AP2REP, Kruppel-like factor 12; OCT1, POU domain transcription factor; ZEB1, zinc finger E-box binding homeobox 1. Click here for file

Additional file 6 Dataset S1: Genomic sequences of intra-*GLI3 *CNEs, tested functionally in transgenic mice assay. Click here for file
