# Supplementary material for: Human intronic enhancers control distinct sub-domains of Gli3 expression during mouse CNS and limb development
Source: BMC Dev Biol. 2010 Apr 28;10:44. doi: 10.1186/1471-213X-10-44 (PMC2875213; doi:10.1186/1471-213X-10-44)
Supplement: Additional file 1 — Table S1: Tetrapod-Teleost Conserved Non-Coding elements (CNEs) from Introns of Human GLI3 Selected for Functional Analysis in Transgenic mice assay. [file 1471-213X-10-44-S1.DOC]

| Table S1. Tetrapod-Teleost Conserved Non-Coding elements (CNEs) from Introns of Human *GLI3* Selected for Functional Analysis in Transgenic Mice Assay | | | | | | | | | |
| --- | --- | --- | --- | --- | --- | --- | --- | --- | --- |
| **Region** | **Element** | **Amplicon Coordinates Chr7** | **Amplicon Size** | **Conservation Human-Fugu 50%; >60 bp** | **Name of construct** | **Number of transgenic mouse lines established** | **Positive for X-gal signal** | **Independent lines showing consistent expression** | **Number of embryos from positive lines showing expression pattern reported in this study** |
| Intron 2 | CNE1 | 42219598- 42220542 | 945 bp | 935 bp | CNE1-prom beta- globin-LacZ | 3 | 2/3 | 2 | 62 |
| Intron 3 | CNE10 | 42125837- 42126969 | 1133 bp | 105 bp | CNE10-prom beta- globin-LacZ | 3 | 1/3 | 1 | 22 |
| Intron 10 | CNE6 | 42018164- 42019025 | 862 bp | 179 bp | CNE6-prom beta- globin-LacZ | 5 | 1/5 | 1 | 32 |
| Intron 10 | CNE11 | 42002211-42003395 | 1185 bp | 129 bp | CNE11-prom beta- globin-LacZ | 6 | 1/6 | 1 | 16 |
| Intron 13 | CNE9 | 41975857 -41976525 | 669 bp | 108 bp | CNE9-prom beta- globin-LacZ | 5 | 2/5 | 2 | 40 |
| Location, size, coordinates (NCBI 36, Oct 2005) of the selected subset of human-*Fugu* CNEs (functionally tested in this study) are indicated. The name of the constructs used to generate permanent transgenic mouse lines for each CNE is given. In addition, the table also provides information about the number of independent transgenic lines established for each construct, proportion of independent lines which were positive for *lacZ* reporter expression, and number of independent lines showing consistent expression pattern in the tissue domains reported in this study. The number of embryos from positive lines that show expression depicted in the Figures (main text) is given. | | | | | | | | | |
