# Supplementary material for: Human intronic enhancers control distinct sub-domains of Gli3 expression during mouse CNS and limb development
Source: BMC Dev Biol. 2010 Apr 28;10:44. doi: 10.1186/1471-213X-10-44 (PMC2875213; doi:10.1186/1471-213X-10-44)
Supplement: Additional file 2 — Figure S1: ClustalW-derived multiple alignment of CNE6 sequence across a diverse set of mammalian species. Star symbols underneath represent nucleotide positions conserved in all species. Conserved putative transcription factor binding sites (TFBSs) are enclosed in rectangles. ALX4, aristaless-like homeobox 4; SOX5, SRY (sex determining region Y)-box 5; PITX2, paired-like homeodomain transcription factor 2; LHX3, LIM homeobox protein 3; HOXD13, homeobox D13; PITX1, paired-like homeodomain transcription factor 1; GLI, GLI family zinc finger; HOXD11, homeobox D11; HOXA7, homeobox A7; dHAND, basic helix-loop-helix transcription factor; MSX1, msh homeobox 1; PBX1, pre-B-cell leukemia homeobox 1. [file 1471-213X-10-44-S2.PDF]

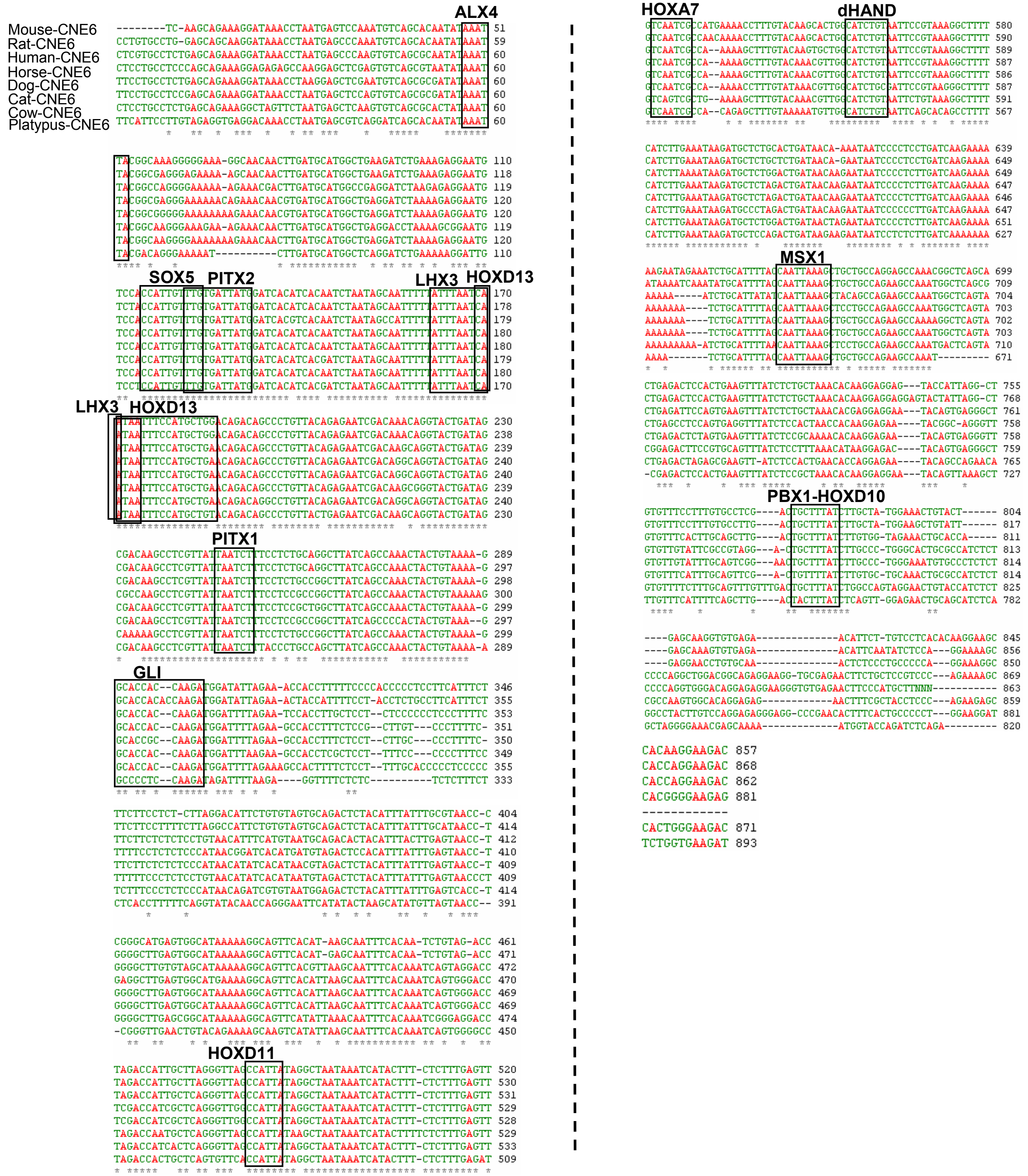
Mouse-CNE6
Rat-CNE6
Human-CNE6
Horse-CNE6
Dog-CNE6
Cat-CNE6
Cow-CNE6
Platypus-CNE6
ALX4
SOX5
PITX2
LHX3
HOXD13
LHX3
HOXD13
PITX1
GLI
HOXD11
HOXA7
dHAND
MSX1
PBX1-HOXD10
