# Supplementary material for: Human intronic enhancers control distinct sub-domains of Gli3 expression during mouse CNS and limb development
Source: BMC Dev Biol. 2010 Apr 28;10:44. doi: 10.1186/1471-213X-10-44 (PMC2875213; doi:10.1186/1471-213X-10-44)
Supplement: Additional file 3 — Figure S2: ClustalW-derived multiple alignment of CNE11 sequence across a diverse set of mammalian species. Star symbols underneath represent nucleotide positions conserved in all species. Conserved putative transcription factor binding sites (TFBSs) are enclosed in rectangles. HOXA13, homeobox 13; HOXD13, homeobox 13; dHAND, basic helix-loop-helix transcription factor; TBX3, T-box 3; HOXA3, homeobox 3; PITX2, paired-like homeodomain transcription factor 2. [file 1471-213X-10-44-S3.PDF]

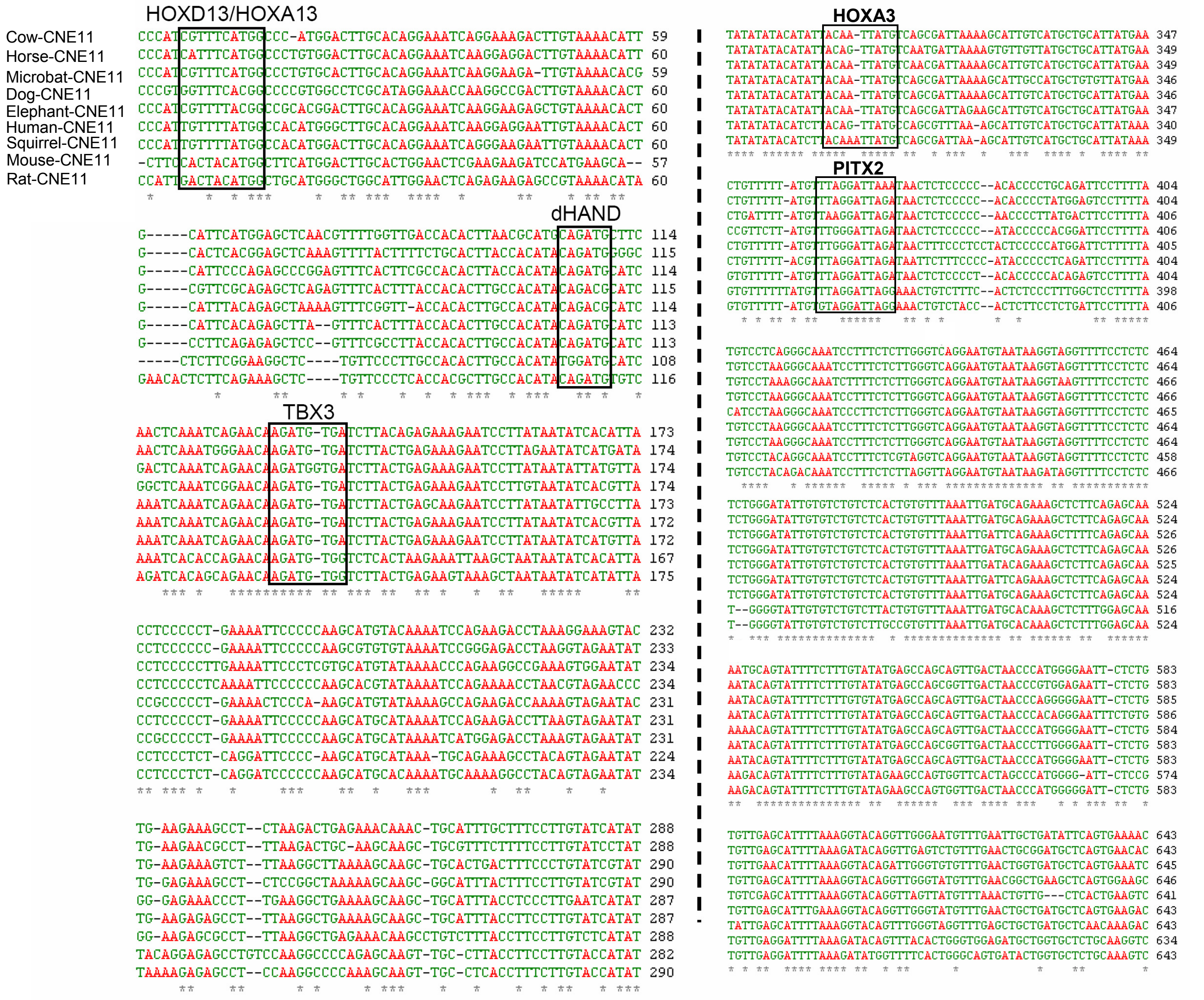

HOXD13/HOXA13
Cow-CNE11
Horse-CNE11
Microbat-CNE11
Dog-CNE11
Elephant-CNE11
Human-CNE11
Squirrel-CNE11
Mouse-CNE11
Rat-CNE11
dHAND
TBX3
HOXA3
PITX2
