# Supplementary material for: Human intronic enhancers control distinct sub-domains of Gli3 expression during mouse CNS and limb development
Source: BMC Dev Biol. 2010 Apr 28;10:44. doi: 10.1186/1471-213X-10-44 (PMC2875213; doi:10.1186/1471-213X-10-44)
Supplement: Additional file 4 — Figure S3: ClustalW-derived multiple alignment of CNE1 sequence across a diverse set of amniotic vertebrate species. Star symbols underneath represent nucleotide positions conserved in all species. Conserved putative transcription factor binding sites (TFBSs) are enclosed in rectangles. MEIS1, Meis homeobox 1; PAX3, paired box 3; FOXM1, forkhead box M1; SOX5, SRY (sex determining region Y)-box 5; HOXA4, homeobox 4; FOXP3, forkhead box P3; dHAND, basic helix-loop-helix transcription factor; MSX1, msh homeobox 1; HOXA3, homeobox 3; NFKB1, nuclear factor kappa-B; GATA3, GATA binding protein 3; CDX2, caudal type homeobox 2; OCT4, POU domain, class 5, transcription factor1; TBX5, T-box 5; CHX10, visual system homeobox 2; SP1, Sp1 transcription factor; PBX1, pre-B-cell leukemia homeobox 1; TCF. Transcription factor; LEF, lymphoid enhancer binding factor. [file 1471-213X-10-44-S4.PDF]

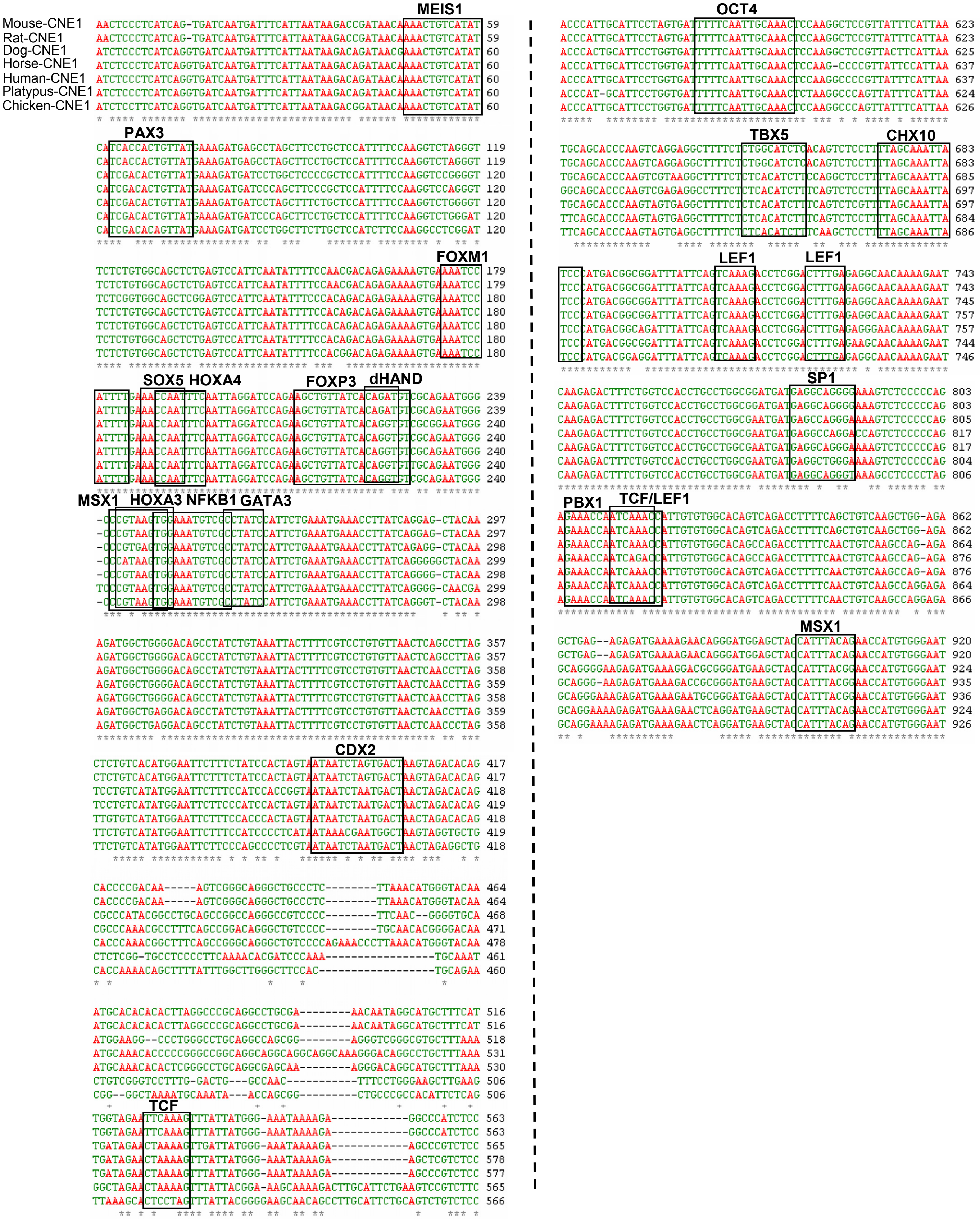
Mouse-CNE1
Rat-CNE1
Dog-CNE1
Horse-CNE1
Human-CNE1
Platypus-CNE1
Chicken-CNE1
MEIS1
PAX3
FOXM1
SOX5
HOXA4
FOXP3
dHAND
MSX1
HOXA3
NFKB1
GATA3
CDX2
TCF
OCT4
TBX5
CHX10
LEF1
LEF1
SP1
PBX1
TCF/LEF1
MSX1
