# Supplementary material for: Human intronic enhancers control distinct sub-domains of Gli3 expression during mouse CNS and limb development
Source: BMC Dev Biol. 2010 Apr 28;10:44. doi: 10.1186/1471-213X-10-44 (PMC2875213; doi:10.1186/1471-213X-10-44)
Supplement: Additional file 5 — Figure S4: ClustalW-derived multiple alignment across a diverse set of amniotic vertebrate species reveals highly conserved putative TFBSs within the core of the CNE9 interval. Star symbols underneath represent nucleotide positions conserved in all species. Conserved putative transcription factor binding sites are enclosed in rectangles. HOXA3, homeobox 3; TBX5, T-box 5; AP2REP, Kruppel-like factor 12; OCT1, POU domain transcription factor; ZEB1, zinc finger E-box binding homeobox 1. [file 1471-213X-10-44-S5.PDF]

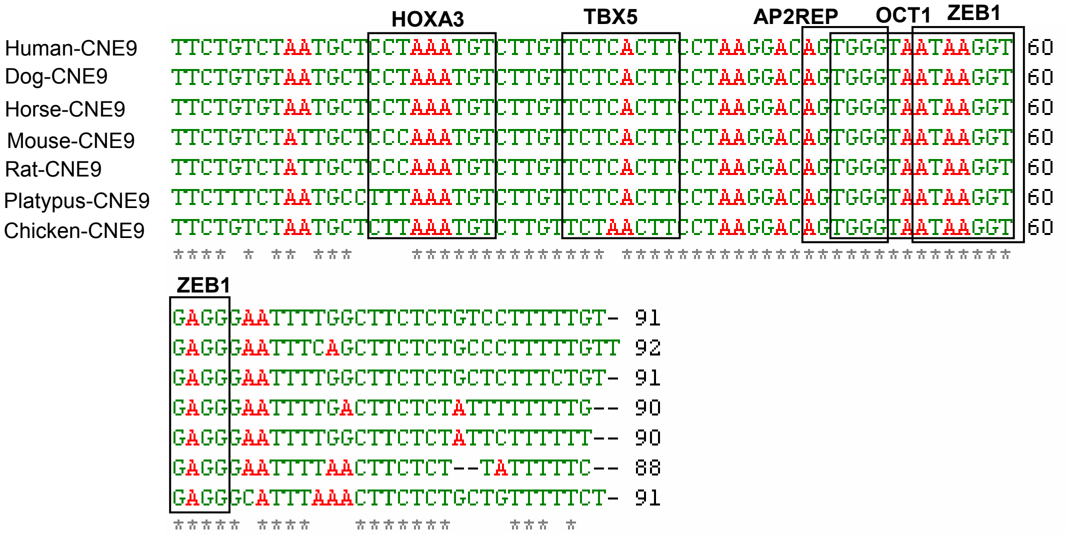
HOXA3
TBX5
AP2REP
OCT1
ZEB1
Human-CNE9 TTCTGTCTAATGCTCCTAAATGTCTTGTTCTCACTTCCTAAGGACAGTGGGTAATAAGGT 60
Dog-CNE9 TTCTGTGTAATGCTCCTAAATGTCTTGTTCTCACTTCCTAAGGACAGTGGGTAATAAGGT 60
Horse-CNE9 TTCTGTGTAATGCTCCTAAATGTCTTGTTCTCACTTCCTAAGGACAGTGGGTAATAAGGT 60
Mouse-CNE9 TTCTGTCTATTGCTCCCAAATGTCTTGTTCTCACTTCCTAAGGACAGTGGGTAATAAGGT 60
Rat-CNE9 TTCTGTCTATTGCTCCCAAATGTCTTGTTCTCACTTCCTAAGGACAGTGGGTAATAAGGT 60
Platypus-CNE9 TTCTTTCTAATGCCTTTAAATGTCTTGTTCTCACTTCCTAAGGACAGTGGGTAATAAGGT 60
Chicken-CNE9 TTCTGTCTAATGCTCTTAAATGTCTTGTTCTAACTTCCTAAGGACAGTGGGTAATAAGGT 60
ZEB1
GAGGGAATTTTGGCTTCTCTGTCCTTTTTGT- 91
GAGGGAATTTCAGCTTCTCTGCCCTTTTTGTT 92
GAGGGAATTTTGGCTTCTCTGCTCTTTCTGT- 91
GAGGGAATTTTGACTTCTCTATTTTTTTTG-- 90
GAGGGAATTTTGGCTTCTCTATTCTTTTTT-- 90
GAGGGAATTTTAACTTCTCT--TATTTTTC-- 88
GAGGGCATTTAAACTTCTCTGCTGTTTTTCT- 91
