# Supplementary material for: Human intronic enhancers control distinct sub-domains of Gli3 expression during mouse CNS and limb development
Source: BMC Dev Biol. 2010 Apr 28;10:44. doi: 10.1186/1471-213X-10-44 (PMC2875213; doi:10.1186/1471-213X-10-44)
Supplement: Additional file 6 — Dataset S1: Genomic sequences of intra-GLI3 CNEs, tested functionally in transgenic mice assay. [file 1471-213X-10-44-S6.DOC]

**Dataset S1**

**Genomic sequences of intra-*GLI3* CNEs, tested functionally in transgenic mice assay**

>CNE1 (945bp)

CTTAGGAGACCATTCCCACATGGTTCCGTAAATGGTAGCTTCATCCCGCATTCTTTCATCTCTTTCCCTGCTCTCTGGCTTGACAGTTGAAAAGGTCTGACTGTGCCACACAATGGTTTGATTGGTTTCTCTGGGGGAGACTTTTCCCTGCCTCATCATTCGCCAGGCAGGTGGACCAGAAAGTCTCTTGATTCTTTTGTTCCCTCTCAAAGTCCGAGGTCTTTGACTGAATAAATCTGCCGTCATGGGATAATTTGCTAAAACGAGACTGAAAGATGTGAGAGAAAAGCCTCACTACTTGGGTGCTGCATTAATGAAATAACGGGGCCTTGGAGTTTGCAATTGAAAAATCACCAGGAATGCAATGGGTGGAGACGGGCTTCTTTTATTTCCCATAATAAACTTTTAGTTCTATCATTTAAAGCATGCCTGTCCCTTTGCTCGCCTGCAGGCCCGAGTGTGTTTGCATTTGTACCCATGTTTAAGGGTTTCTGGGGACAGCCCTGCCCGGCTGAAAGCCGTTTGGGTGCTGTGTCTAGTTAGTCATTAGATTATTACTAGTGGGTGGAAAGAATTCCATATGACACAACTAAGGTTGAGTTAACACAGGACGAAAAGTAATTTACAGATAGGCTGTCCCCAGCCATCTTTGTAGCCCCTGATAAGGTTTCATTTCAGAATGGATAGGCGACATTTCCACTTACGGGCCCATTCTGCGACACCTGTGATAACAGCTTCTGGATCCTAATTGAAATTGGTTTCAAAATGGATTTTCACTTTTCTCTGTCTGTGGAAAATATTGAATGGACTCAGAGCTGCCACAGAGAACCCCAGACCTTGGAAAATGGAGCAGAAAGCTAGGATCATCTTTCATAACAGTGTCGATGATATGACAGTTTTGTTATCTGTCTTATTAATGAAATCATTGATCACCTGATGAGGGAG

>CNE2 (434bp)

GAGCAATTGCAGAGTGCAGGCCGGTTAAGGGATTGAGCTATATGCACTATTATTGCAAGAAGTATTCCGAAATACCAGAAATAGGACGTAAGCTCTGATCAGGGAGACTGCGAGCACAATTACCTTCTTTTCAAATCCTTCTGTGACACTGCGGGAGGAAAAAGGACTTTGAAACTTGAAAGGAAAGAGCTTGCTTTCAACCTCAAAAGCTAGGAGGAAAGGGCTCTGAAATTTGCTCAGAATTCCCAATTCACCATTAGCCTGTTTCTTCCTTTAGCCTCAAGGCATTCTCCGCTTTTTGAAAAGATGTTAAGAAATTCAGTCACAATAGAGAGCCTAGTTTTGAACATGTTTCACTCGGTCCATTGAGGTCTAGGCTCCAGCCTTTGTGTGGGGTGAATTGAGCTGAGCGGCTAGCTGGTTGGAGAGAGGTG

>CNE6 (862bp)

CTCGTGCCTCTGAGCAGAAAGGATAAACCTAATGAGCCCAAGTGTCAGCGCAATATAAATTACGGCCAGGGGAAAAAAGAAACGACTTGATGCATGGCCGAGGATCTAAGAGAGGAATGTCCACCATTGTTTGTGATTATGGATCACGTCACAATCTAATAGCCATTTTTATTTAATCAATAATTTCCATGCTGAACAGACAGCCCTGTTACAGAGAATCGACAAGCAGGTACTGATAGCGACAAGCCTCGTTATTAATCTTTCCTCTGCCGGCTTATCAGCCAAACTACTGTAAAAGGCACCACCAAGATGGATTTTAGAATCCACCTTGCTCCTCTCCCCCCTCCCTTTTCTTCTTCTCTCTCCTGTAACATTTCATGTAATGCAGACACTACATTTACTTGAGTAACCTGGGGCTTGTGTAGCATAAAAAGGCAGTTCACGTTAAGCAATTTCACAAATCAGTAGGACCTAGACCATTGCTCAGGGTTAGCCATTATAGGCTAATAAATCATACTTTCTCTTTGAGTTGTCAATCGCCAAAAAGCTTTGTACAAGTGCTGGCATCTGTAATTCCGTAAAGGCTTTTCATCTTAAAATAAGATGCTCTGGACTGATAACAAGAATAATCCCCTCTTGATCAAGAAAAAAAAAATCTGCATTATATCAATTAAAGCTACAGCCAGAAGCCAAATGGCTCAGTACTGAGATTCCAGTGAAGTTTATCTCTGCTAAACACGAGGAGGAATACAGTGAGGGCTGTGTTTCACTTGCAGCTTGACTGCTTTATCTTGTGGTAGAAACTGCACCAGAGGAACCTGTGCAAACTCTCCCTGCCCCCAGGAAAGGCCACCAGGAAGAC

>CNE9 (669bp)

CACATTTCCACAGCAGATATTATGTGTGTATATGCTCACACACATTTAAAAGTAGGTATCTTCACAGACATTTACTAATAAACATGAAATTTACATTTCTACTTTTCTTTAAAAAACATGTCTTTGATTTGATCATCATGCTATAAATTAAATTCTTTCTTCTTTTTTCTTATTAATGCAACATCTCTTCTGTCTAATGCTCCTAAATGTCTTGTTCTCACTTCCTAAGGACAGTGGGTAATAAGGTGAGGGAATTTTGGCTTCTCTGTCCTTTTTGTTTTGTTTTCATTTTTTTAACATTTACTTTTTTCTGTTAATTTTTCTTTAGTTTTTGTGGTTTTGTGATACTTTTGGTAGTGAACATTACTCAGATATGTTTCAAATATAGAAGCAATGTGTTTTCAACATTCAGCATGTGAAAAATGTGGAGTTAGTTGGTGAAATTCATTGTTCTATCTGTCTAGGTTTCCATAACAAATCACACTGGGGCCTACCAAGCAGATGTTCTGAAAGACTCATTATTGTACTGACTAATGCAAATTCATCCTTAGACCAGATGTGCACCACCTTCTTTGAGGAAGATCTGGCTTTATTTGTTGTTAGCAATGTAATCTCTATTTAAATGTGGACGCTCATTGAAGCAATTTCCTAATTGTGAGTGCATTTGTG

>CNE10 (1132bp)

CAATCAGCAGAAATCTACTAAATCCCTCTTAAAAGGTCCATTCTAAACAACATTCCACAGTGACACAGGGGCCTGACTTTGATTTCTGAATAATTTGACTAGAAGCAAGGAAAGTCACAACAACATTAAGTGCAATAACTTTCTACCCCCTGTTTGGTTCCCAGTGTGAAATTTGCTGAAATAAAACTTGACAGCCCAAAACTCCCGGGGATGTGTTCCAGATGAATCGAGTCATCCACACAAACTCACAGACACACGCACACACGCCCTGGACCCTCGCACTTATAGAGGGAGTTGTCTCCATCTGTATTTGACAACCCGGTGCCATGGGTCTCAAGGCTGAGGGCTGCTTATGGATGCAACTTTAAATTTAATTTGCGGGGGATGATAATTGTGTGGAGTGGTAGGAAAATGCTGGCGACATTTTGACTTTAGCAGCCCCACTGCCCTGATTAAAAAATATCAGCTTTCCATATGCAAGACATTTCTAAGGTGGACACCTTACAAATTGTTAAGGTGCAATTTGTGCACAGTTAGTTCTTTGTTTAATCGAGAAATCTGCCCGAACTCTGATTGGGCTGATTTCTCTAAGGCCGGGAGTGAAGGTTATTTCCTTATGCCTTTTGGGAGCAGCAATCTCTCTAATACGTTTCTGAATTATTCCAGCTTGTGCCGAGGTTGAACAGGAGAACAAAGCCCTGGTTCCACAGCAGAAAGACTAACTGAACTCCCAGCTGGGAAGGTTTAGGCAGAGATTTATAAGTACAAACACTGAAGAAATTTGCAAGTAATTGCATGTATTTGCTTTCCGTTGTGGGATGCAGTAAACCAGTATTTATTAAAATGCATCGGCGAGGTACTGTTTATTACCCCCTCTCCTTGATGTCTTGTAGAGGGGAATAAAGTGCTTGGTTCTTTTCATTTCTTTAAATTAAGGGGGCAAAACAGTATTTCAAAGAATATAAGGAAGAAGCTGAAATTAGACATATACTCTGAATGGCTACTGCCTCTTACTTAACTTGGAATTGGTGCATGAACATGGCTCCAGATATGCTGTTCTGATTTAATCTTGTAAAGCTTAACTTTTTTTCTACATTCATAAGTTTCCATGAATCCAGTATTTGGGGATCAAC

>CNE11(1185bp)

CTGGACTTCTCAAAATGTCAGGTTTCTTTATCAGCCTTCCAACCAATCACTCAGGGATGCCTTTCCTTCCTGGGAGATGGTACCATAGGCTTACATTATATCATGTATGGAAACATCTGGGGGAAAAACTCAACTCCCATTAAAAAATATATTTTATATAGCCCTGAAAGGAAAACAGTGAACGCCCACACCACTCACAGCTGAGATTACGGGAGTGTCTCTCCCAGCAGCTTTCCCATATGAAGAAATAAAAATCCCAATTCTTCTGCCCATTGTTTTATGGCCACATGGGCTTGCACAGGAAATCAAGGAGGAATTGTAAAACACTGCATTCACAGAGCTTAGTTTCACTTTACCACACTTGCCACATACAGATGCATCAAATCAAATCAGAACAAGATGTGATCTTACTGAGAAAGAATCCTTATAATATCACGTTACCTCCCCCTGAAAATTCCCCCAAGCATGCATAAAATCCAGAAGACCTTAAGTAGAATATTGAAGAGAGCCTTTAAGGCTGAAAAGCAAGCTGCATTTACCTTCCTTGTATCATATTATATATACATATTACAATTATGTCAGCGATTAAAAGCATTGTCATGCTGCATTATGAACTGTTTTTACGTTTAGGATTAGATAATTCTTTCCCCATACCCCCTCAGATTCCTTTTATGTCCTAAGGGCAAATCCTTTCTCTTGGGTCAGGAATGTAATAAGGTAGGTTTTCCTCTCTCTGGGATATTGTGTCTGTCTCACTGTGTTTAAATTGATTCAGAAAGCTCTTCAGAGCAAAATACAGTATTTTCTTTGTATATGAGCCAGCGGTTGACTAACCCTTGGGGAATTCTCTGTGTTGAGCATTTGAAAGGTACAGGTTGGGTATGTTTGAACTGCTGATGCTCAGTGAAGACCACTGGGAAAGTGGATTGGCCGTGGCTCTGAGAGAGGCCTTTTCTCTAATGTGGACTCGAGGAAAAAAAAAATAATTGTTCCAACAAATAATTTTTAATGGTACAATAATTTAGGAAAATTAGCCAGTTCATTTTAATAAGAATGGATCAAGACCAAAATAAAGAGGGAATTATAGTTTATACAAATGTATGAACATACAGTTTAAAGGCATTTAGCCACCAAAAATAAATTTTCTAAAAACAGCAGTTTTCTCTCCATCCACTGTATGAAGTG
